# Cellular senescence is a key mediator of lung aging and susceptibility to infection

**DOI:** 10.3389/fimmu.2022.1006710

**Published:** 2022-08-31

**Authors:** Blake L. Torrance, Laura Haynes

**Affiliations:** UConn Center on Aging and Department of Immunology, School of Medicine, University of Connecticut, Farmington, CT, United States

**Keywords:** senescence, influenza, lung, aging, immunity

## Abstract

Aging results in systemic changes that leave older adults at much higher risk for adverse outcomes following respiratory infections. Much work has been done over the years to characterize and describe the varied changes that occur with aging from the molecular/cellular up to the organismal level. In recent years, the systemic accumulation of senescent cells has emerged as a key mediator of many age-related declines and diseases of aging. Many of these age-related changes can impair the normal function of the respiratory system and its capability to respond appropriately to potential pathogens that are encountered daily. In this review, we aim to establish the effects of cellular senescence on the disruption of normal lung function with aging and describe how these effects compound to leave an aged respiratory system at great risk when exposed to a pathogen. We will also discuss the role cellular senescence may play in the inability of most vaccines to confer protection against respiratory infections when administered to older adults. We posit that cellular senescence may be the point of convergence of many age-related immunological declines. Enhanced investigation into this area could provide much needed insight to understand the aging immune system and how to effectively ameliorate responses to pathogens that continue to disproportionately harm this vulnerable population.

## 1 Introduction

With age, there are many changes that occur throughout the body and affect nearly every organ system, including the respiratory and immune systems. The primary function of the lungs is to facilitate effective gas exchange, but another critical function is serving as a mucosal barrier site that is adequately able to resist infection despite being exposed to a legion of microbes and potential pathogens on a daily basis. The complex organization of various tissues and cell types in the lungs, non-immune and immune alike, are responsible for coordinating these important functions. Aging will affect all facets of this system and will go on to cause age-related dysfunction both in the normal physiological function of the lungs as well as leaving the host at great risk for infections and subsequent morbidity and mortality. While there are many processes of aging that can cause dysfunction in these areas, our focus in this review is the contribution of cellular senescence and the accumulation of senescent cells in causing and exacerbating age-related declines in the lungs.

Cellular senescence is characterized by irreversible growth arrest that occurs when cells experience a stressor (1). Senescence has two main roles in an adult: one is to suppress cells that have incurred DNA or other damage to prevent them from transitioning into cancer (2), the other is to aid in wound healing (3). The stimuli that lead to senescence are well reviewed elsewhere (4), but include DNA damage, replicative exhaustion, oxidative or metabolic stress, oncogene expression, phototoxic stress, shear stress, as well as inflammation. The key mediators of the cell cycle arrest include p21^Cip1^ (p21) and p16^INK4A^ (p16), which are cyclin dependent kinase inhibitors (5). Often, expression of these molecules is used as a marker for senescence but they should be combined with other assays for DNA damage, telomere shortening, or senescence associated beta-galactosidase activity to definitively conclude that a cell is senescent. Importantly, after senescent cells perform their physiological functions, they are typically cleared by the innate immune system, including macrophages and natural killer (NK) cells (6). Effective clearance is necessarily reliant on a well-functioning immune system and therefore, with age, senescent cells are more likely to evade clearance due to age-related changes in the immune system.

When senescent cells fail to be detected and cleared, they begin to cause dysfunction. Senescent cells engage various pathways to enforce resistance to apoptosis *via* a variety of pathways as reviewed by others (7), collectively termed senescent cell anti-apoptotic pathways (SCAPs). Engagement of SCAPs furthers the accumulation of these cells, which can have deleterious effects on the microenvironments where senescent cells are found and beyond. Aside from the cell-intrinsic dysfunction of senescent cells, a key hallmark of senescence is the secretion of a generally-proinflammatory heterogenous cocktail of soluble factors including cytokines and chemokines, termed the senescence associated secretory phenotype (SASP). Not only does this alter the cytokine composition of the microenvironment which will disrupt physiological cell signaling networks, but it can be a source of inflammation resulting in the induction of senescence in nearby cells in a paracrine manner (8). This cycle of senescence induction can wreak havoc on dynamic and cell-rich tissues, such as those that comprise the respiratory system. In this review, we aim to highlight work that links cellular senescence and chronic diseases of aging in the lung as well as the ability of the immune system to adequately respond to respiratory infections.

### 1.1 Effects of aging and senescence on development of chronic lung pathologies

In order for the lungs to function properly, many different tissue structures, cell types, signaling mechanisms, and other processes need to be tightly regulated. Air is conducted through the trachea and branches into bronchi that infiltrate into the lungs and continue to branch into smaller bronchioles that terminate in alveoli wherein gas exchange occurs with blood vessels in close association. A complex community of cells facilitates these processes at each distinct tissue region. In the bronchi, goblet cells secrete mucus that trap debris and potential pathogens while ciliated cells work to move the mucus and its contents up and away from the lower respiratory tract. Found in the alveolus, type I and II pneumocytes are specialized epithelial cells that line the majority of the alveolar space to perform gas exchange or secrete necessary surfactants, respectively. Nearby, endothelial cell tight junctions in the blood vessel achieve limited permeability to protect the airway from blood infiltrate. Fibroblasts are also present in the alveolus and provide important structural and barrier integrity functions. Various resident immune cells are also found in the airway during homeostasis (in the absence of infection), notably alveolar macrophages that surveil the airway and sample incoming antigens taken in from the airway. Two major chronic diseases of aging, chronic obstructive pulmonary disease (COPD) and idiopathic pulmonary fibrosis (IPF), have been linked to cellular senescence. Here, we briefly discuss recent efforts linking COPD and senescence. IPF has been more definitively shown to be primarily driven by senescence (9) *via* similar mechanisms and are comprehensively reviewed elsewhere (10). Further, targeting senescence in patients with IPF has already been shown effective in an early stage clinical trial (11). Both pathologies serve as a case in point for our discussion of mechanisms by which senescence can cause baseline dysfunctions in the respiratory system that can be a detrimental risk for developing severe respiratory infections.

#### 1.1.1 Senescence and COPD development

Chronic obstructive pulmonary disease (COPD) is a chronic illness that can occur as a result of chronic bronchitis and/or emphysema. Induction is typically caused by chronic exposure to cigarette smoke or other toxins. Bronchitis is characterized by increased inflammation of the bronchi while emphysema is characterized by severely damaged alveoli (12). Common symptoms include shortness of breath, persistent cough, overproduction of mucus, and wheezing. While the majority of COPD patients can be linked to smoking, aging processes in the lung, particularly cellular senescence, certainly accelerate the development of the disease. For years, COPD has been described as a disease of accelerated aging in the lungs (13). The exact contribution of age versus environmental exposure to the development of COPD is not entirely clear. There are a substantial number of early-onset COPD patients who develop symptoms under the age of 53 and often are not heavy smokers (14). The etiology of these age-independent cases of COPD has remained elusive. However, recent work has pointed towards cellular senescence a driver of COPD onset and severity.

A number of hallmarks of senescence can be identified in COPD development. Increased inflammatory cytokine and chemokine expression is commonly observed (15). IL-6 and other molecules related to IL-6 signaling are among the most consistent (16, 17). Interestingly, IL-6 is also among the most consistent factors present in the SASP of senescent cells (18). Mouse models of cigarette smoke induced COPD have indicated smoke-induced senescence as a mediator of pathogenesis (19). More recently, the first concrete link between senescence and COPD was described by demonstrating a greater frequency of senescent lung fibroblasts from COPD patients (including early onset) compared to non-COPD controls (20). This study utilized expression of both p21 and p16, γ-H2A.X (DNA damage marker), and SA-β-Gal. Fibroblasts in the lung are principally associated with mediating extracellular matrix (ECM) remodeling, as reviewed elsewhere (21). Increased secretion of TGF-β by fibroblasts been associated with COPD development as well (22). When senescence occurs in these cells, it can cause aberrant repair mechanisms similar to a wound healing or tissue remodeling response. These responses have been long since linked to senescence and the SASP (23), including enhanced TGF-β production. Clearly, this indicates that senescent cell participation can lead to COPD, but these same mechanisms can also severely disrupt the ability of the immune system to respond effectively to an incoming pathogen.

Disruptions of basal cytokine signaling will certainly alter the response of immune cells. Increased production of SASP factors can also alter the composition of the immune cell compartment in the lungs. It has been reported that increased chronic inflammation with age, likely exacerbated by SASP, results in a change in homeostatic CD4+ T-helper cell (Th) subset balances. Notably, there is a shift towards circulating IL-17 producing Th17 cells over regulatory T cells (Tregs) with age (24). This same Th17/Treg ratio dysfunction has also been linked to COPD (25). Dysfunctional cytokine signaling will also alter the responsiveness of innate immune cells like alveolar macrophages, dendritic cells, and neutrophils, all of which are critical first line responders to respiratory pathogens. Therefore, mechanisms of senescence highlighted by chronic disease development can alter the function of both non-immune and immune cell compartments in the absence of an infection, which will have severe consequences when a pathogen enters and requires a highly coordinated response to effectively control.

## 2 Aging and senescence in regulating immune responses to infections

Chronological aging is associated with a wide-ranging decline in overall efficacy of the immune response. Unfortunately, the COVID-19 pandemic has made this fact abundantly clear. At the time of preparing this submission, over 700,000 people aged 65 or older have succumbed due to COVID-19 infection in the United States alone (26). Our group and others have extensively studied the effects of age on responses to influenza (flu) infection, which similarly is a disproportionate concern for individuals over 65. This population, on average, accounts for 70-85% of flu-related deaths each season (27). These vulnerabilities are mostly caused by deficits in the immune system and the ability to mount an appropriate response to an infection. Much work has been done characterizing the changes in the immune system associated with chronological age, but we wish to highlight and discuss a few key points.

### 2.1 The innate immune compartment

Chief among the age-associated changes in the immune system is the increase in chronic, low-grade, sterile inflammation. This phenomenon, termed inflammaging, was originally described a number of years ago (28–30) and has been broadly defined as increased systemic and local concentrations of pro-inflammatory cytokines including IL-6, TNF-α, IL-1β, and others. Aside from influencing the tightly regulated immune responses to pathogens, acute inflammation and inflammaging has been associated with the progression and development of many diseases of aging including Alzheimer’s disease (31), type 2 diabetes (32), and osteoarthritis (33). Interestingly, some studies focusing on healthy older adults have found no increased systemic IL-6 or TNF-α in their cohorts (34, 35). Utilizing a sophisticated computational deep-learning model, CXCL9 was identified as a more informative biomarker for inflammaging (35). The exclusion criteria of these studies were relatively strict compared to other studies and perhaps this can explain some of the discrepancy. It is plausible that senescent cell burden may influence the degree of inflammaging across individuals due to SASP production. Further, this may highlight the pathogenic role of these cytokines in developing chronic diseases of aging. The source of this systemic accumulation of cytokines has been an area of active research and has yielded a wide spectrum of cell types and signaling mechanisms that could be the source this inflammation. Considering the pro-inflammatory phenotype exhibited by senescent cells, they are a major contributor to inflammaging, however, age-related changes in innate immune signaling are also responsible for a large portion of this phenomenon.

Toll-like receptor (TLR) stimulation undergoes changes with age. In general with aging, TLRs are subject to more chronic stimulation, perhaps through increased leaking of microbial products from the microbiome, which has been demonstrated in older adults (36) and aged mice (37). Chronic stimulation of TLRs in both immune and non-immune cells will engage canonical cytokine signaling mechanisms through NF-κB, which is also known to be hyperactive with aging (38). Hyperresponsiveness to pathogen-associated molecular patterns (PAMPs) *via* commensal microbial products will impair the ability of both infected cells and cells of the innate immune system to appropriately respond to invading pathogens. Importantly, for reasons we will discuss in depth later, pathogen clearance is impaired with aging. This will result in lingering stimulation of TLRs which then leads to persistent cytokine secretion and enhanced recruitment of phagocytic cells. This initiates a vicious cycle of abundant cytokine production, which impairs the ability to resolve the infection and the return to homeostasis. While these mechanisms can impact the function of all innate immune cells, the effects of aging on antigen presenting cells (APCs) can exacerbate dysfunctions in the adaptive arm of the immune system as well.

When mounting a response to a pathogen in the respiratory tract, dendritic cells (DCs) are the key nexus of innate-adaptive immune crosstalk. Aside from participating in and being affected by increased tonic cytokine signaling, DCs perform an integral function in coordinating the activity of first line innate defenses and the robust response of T and B lymphocytes. While a number of mechanisms have been identified by which age affects DCs, it is still unclear how exactly the overall state of DC function changes with age.

Similar to other innate cell types, DCs from older adults have a propensity to secrete proinflammatory cytokines tonically in the airway (39). This chronic inflammation will leave an individual at higher risk for developing chronic conditions, as discussed earlier, but it will also disrupt the threshold of inflammation necessary to induce a robust immune response. Because the baseline levels of inflammation in the airway are increased, it will require higher levels of newly produced proinflammatory cytokines to create the acute spike of inflammation required to kickstart the infiltration of phagocytes and T cells to initiate pathogen control. One of the most important roles of the DC during an infection is their ability to upregulate MHC class II and costimulatory molecules in order to provide a strong signal 1 and 2, priming and costimulation, respectively, to CD4 T cells. This costimulation is typically delivered *via* enhanced surface expression of CD40 and CD80 which will bind to their ligands on the T cell within the context of the immune synapse. In mice, aging has been demonstrated to decrease expression of MHC II as well as costimulatory molecules upon activation (40, 41). However, there exists some cross-species discrepancy when DCs isolated from older adults are analyzed along the same lines: human DCs, even in advanced age, have not been observed to have these specific declines (42–44). It is likely that these older studies were unable to differentiate between the many DC subsets that have been identified and this particular question regarding the effects of age on DC presentation to and costimulation of T cells merits further examination.

The effect of cellular senescence on the function of other cells of the innate immune system with age remains unclear. It has been shown that macrophages can upregulate senescence markers (45) and recent work has linked senescent macrophages to the development of pulmonary fibrosis (46), the disease most intimately linked to cellular senescence. Type II pneumocytes have also been shown to take on a senescent phenotype and can be effectively cleared by ABT-263, a senolytic drug targeting SCAP pathway mediator Bcl-xl (47). Some of these studies utilized a radiation model of inducing senescence, which can be clinically relevant for studying the effects of certain cancer treatments on respiratory function. However, they may not fully capture the landscape of naturally accumulation of senescent cells with chronological age or exposure to common environmental stimuli. Airway fibroblasts, epithelial, and endothelial cells are the most common senescent cell types identified in the lungs (48–51). Because many of these cells are integral for the migration of dendritic cells between the airway and the lymphatics, increased senescent cell burden could be the explanation for the well documented inability of DCs to migrate appropriately with age (43). Increased expression of prostaglandins with age from lung endothelial cells (52) has been demonstrated to impair the lymph node migration of DCs in pulmonary infections (53) and it is possible that increased senescence in the endothelium may be responsible for this phenomenon. Typically senescence causes a change in cell morphology (1), which can disrupt the tight barriers found between cells in the airway among fibroblasts, epithelial, and endothelial cells. Although not yet directly studied, it is likely that senescence could increase permeability and leaking of signaling molecules across disparate tissues, causing dysfunction in cell-to-cell communication. There remains much to uncover in the relationship between cellular senescence and age-associated declines in the innate immune system, it is clear that increased senescent cell burden can influence the function and migration of these cells and alter their ability to respond effectively to a given pathogen.

### 2.2 The adaptive immune compartment

The effects of age on the adaptive arm of the immune system, namely T and B cells, have been extensively studied. These effects range from cell intrinsic changes as well as cell extrinsic environmental influences. The direct interactions between senescence and T and B cell function are rapidly evolving, drawing upon work by our group and many others currently focusing on this problem. Here, we aim to highlight some of the most important age-related changes to T and B cells during homeostasis and how they leave the host unprepared for a pathogen encounter.

#### 2.2.1 B cells

B cells are affected by age across their developmental lifespan. Starting with their generation in the bone marrow, it has been appreciated for nearly 30 years that hematopoiesis of B cells declines with aging (54). Interestingly, this does not result in an overall decrease in frequency of B cells or any subset in the circulation of older adults (note that these types of human studies are limited to the periphery) (55). While the frequency of various peripheral B cell subsets may not be altered, B cells in older individuals remain ill equipped to respond robustly to an infection.

In order for B cell activation to occur, the B cell receptor (BCR) repertoire of naïve B cells must be diverse enough to capture a wide variety of epitopes to initiate a broad and effective antibody response. In recent years, the technology available to comprehensively study the diversity of these receptors, as well as those of T cells, has advanced considerably. Analyses of BCR repertoires taken from lymphoid tissue biopsies of older adults has revealed that blood and lymph node BCR repertoire diversity is markedly lower than that found in young adults (56).

Aside from repertoire diversity, B cells employ affinity maturation *via* somatic hypermutation (SHM) of immunoglobulin variable region genes to fine tune antibody avidity and thus enhance the ability of an antibody to bind to its specific antigen. This process is critical for generating antibodies that have potent neutralizing activity, especially when it comes to stopping the spread of viruses and other pathogens. This process is tightly regulated: upon recognition of cognate antigen by the BCR in a secondary lymphoid organ, along with help given by T follicular helper cells (Tfh), B cells can form germinal centers which are highly organized structures in which BCRs undergo affinity maturation and selection, resulting in antibodies with enhanced antigen binding capacity. SHM is the process that introduces mutations in immunoglobulin variable region genes to achieve this higher binding affinity. In order to perform SHM, B cells must upregulate activation-induced cytidine deaminase (AID) which actually executes the DNA modification (57). It has been observed that, with age, murine B cells are unable to robustly express this key enzyme, causing deficits in SHM and affinity maturation (58). This was also observed in B cells isolated from older adults (59). These enzymes are also involved with class-switch recombination (CSR), which is the means by which B cells which express one immunoglobulin isotype (such as IgM) can switch to express another isotype (such as IgG or IgE). As with SHM, this process is chiefly mediated by activity of AID and is similarly affected by age when AID expression is decreased upon activation. Thus, three key features of B cell responses are impacted by aging: repertoire diversity, affinity maturation, and isotype class switching. Taken together, this results in age-related reductions in humoral responses.

We have mentioned a number of instances where aging, perhaps exacerbated by accumulation of senescent cells, results in tissue disorganization and changes in morphology of critical structural cells. The same applies to the germinal center. The germinal center is composed to two regions: the dark zone, in which SMH and rapid proliferation occurs, and the light zone where B cells interact with antigen-presenting follicular DCs and follicular helper T cells and the highest affinity BCRs are selected for. B cells will cycle from the dark to light zones for a number of rounds until a high affinity BCR is generated. Because these mechanisms are disparately compartmentalized, any disruption in the boundaries can be disastrous. Surrounding the germinal centers in the spleen, the marginal zone is the protective barrier between the rapidly proliferating B cell follicle and the rest of the organ. Expression of cell adhesion molecules along the sinus of this compartment is decreased with age, causing disruptions in trafficking and sequestration of B cells and cells involved with the germinal center reaction (60). Work in our lab demonstrated that the delineation between the disparate B and T cell zones in the spleen is also compromised (61). Recently, a similar study found that stromal cells expressing cell adhesion molecules in the lymph nodes are dysfunctional with age and result in poor germinal center formation and function (62). It is possible these cells may be undergoing cellular senescence which may explain their altered expression levels of cell adhesion molecules. Senescence can alter the morphology of these stromal cells, which may compromise the architecture of these tightly regulated structures. Even if the stromal cells themselves do not become senescent, the altered functionality and basal inflammaging certainly would contribute to their dysfunction. Some reports have even concluded that B cells can take on a senescent phenotype themselves, including SASP factor production and an altered metabolic state (63, 64). Importantly, there remains much to be discovered in order to understand how B cells age, whether senescence can alter their functionality intrinsically, and how this relates to their decreased function with age.

One particular subset of B cells entirely unique to aging has been identified in both mice (65) and humans (66). These age associated B cells (ABCs) are generally proinflammatory and are a subject of great interest in recent years. Since their discovery, they have been most greatly associated with increased incidence of autoimmunity (66, 67) and chronic viral infections like HIV (68). ABCs are more innate-like and typically are activated *via* TLR signaling (typically TLR7 and 9) independent of their BCR and express the transcription factor Tbet, which is typically expressed by antiviral Th1 CD4 T cells (69). ABCs are often functional in the context of antiviral responses and are able to strongly activate T cells and generate highly specific antibodies in response to infections (70). These cells represent an altered functionality of immune aging as opposed to declines. Much work is needed to understand how these cells emerge and the various ways they contribute to immune responses.

#### 2.2.2 T Cells

T cells are also affected by aging in a wide variety of cell intrinsic and extrinsic ways. These cells are primarily responsible for pathogen clearance, especially in dynamic mucosal sites like the respiratory system. Prior to an infection or vaccination, naïve CD4 and CD8 T cells circulate through the lymphatics awaiting activation from an APC, typically a DC. This activation requires DCs to have been adequately polarized to deliver appropriate costimulation to the T cell, a key factor we have already discussed as being less effective with age. Increased inflammaging and circulating SASP from nearby senescent cells will also disrupt “signal 3” of T cell activation, the cytokine microenvironment. After activation, most T cells will respond to the infection and then die while a small percentage will become long lived memory cells that can respond better and faster upon a subsequent encounter with the same antigen. From the start of activation, aged T cells are at a disadvantage for responding appropriately. However, there are notable intrinsic defects that are also important to note.

Similar to the B cell compartment, naïve T cell output sharply declines throughout life, reaching its apex around puberty and decreasing thereafter, occurring concomitantly with thymic involution (71). By age 75, nearly all naïve T cell output is halted entirely (72). The diversity of the T cell receptor (TCR) also undergoes rapid changes with age, with some studies characterizing a general shrinking of the overall diversity, as reviewed elsewhere (72). Many of the early studies relied on flow cytometric analysis of various T cell receptor isoforms. While this approach was capable of detecting broad differences with age, it was not able to comprehensively describe the antigen specificities affected by these differences. Other studies have found that the overall diversity of human TCRs are altered but not in terms of diversity, concluding that older adults can retain highly diverse repertoires (73). These authors suggest that the common finding of reduced diversity may be influenced by perturbation in relative abundance of certain clones, which can be delineated using deeper sequencing methods. In recent years, sophisticated sequencing technologies have begun to tease apart more detailed and nuanced differences revealing that CD4 T cells tend to increase the frequency of shared specificities with age (74). This indicates that aged CD4 T cells are predisposed to respond to a more limited pool of antigens. Interestingly, this phenomenon was not observed in the CD8 compartment. These studies reveal that there may be more nuances to how aging impacts certain aspects of TCR diversity, which have yet to be fully connected to effects on the functionality of responses in older populations.

While age limits the capability of T cells to recognize antigen, it also affects the functionality of these cells upon their activation. We first turn to the CD4 compartment which is indispensable in offering “help” in the form of cytokines to CD8 T cells and B cells. CD4 T cells recognize antigen in the context of MHC class II expressed on DCs (and other APCs) and then they differentiate into T helper subsets depending on the cytokine environment. The goal of this differentiation is to generate T helper subsets that are best suited to combating certain classes of pathogens (for example: Th1 for viral infections and Th2 for parasitic infections). In the absence of infection, age causes dramatic shifts in CD4 subset activity during homeostasis. As mentioned previously, circulating CD4 T cells in older adults take on a pro-inflammatory IL-17-producing Th17 phenotype (24, 75). Th17 cells can be pathogenic in many autoimmune diseases and studies have linked this Th17 induction with osteoporosis in mice and humans (76, 77) and mouse models of colitis (78). Interestingly, recent evidence has emerged pointing towards a role for senescent cells in driving Th17 activity. In a model of osteoarthritis, a study demonstrated that increased senescent cell burden caused naïve CD4 T cells to skew towards a Th17 phenotype which then increased senescent cell burden in a feedforward loop (79). This work raises some important questions, including whether this mechanism can be applicable in other situations. If so, it may provide interesting therapeutic strategies to diminish the effects of senescent cells and rescue CD4 T cell subset balance in the absence of infection. This also suggests that treatment with IL-17 inhibitors may mimic the effects of senolytics, which are drugs that clear senescent cells from the body. Future studies elucidating these points may prove fruitful as the field searches for interventions that can rejuvenate T cell function with age.

Regulatory T cells (Treg), expressing the transcription factor FoxP3, also undergo complex changes with age, perhaps exacerbated by SASP. Canonically, Tregs are important for homeostasis by enforcing tolerance to self-antigens and preventing improperly robust responses to innocuous antigen (80). A subset of Tregs develop naturally from the thymus (nTregs) and are thus affected by thymic involution and declines in T cell production like their conventional T cell counterparts. In mouse models, it has been demonstrated that Treg production actually declines faster and more severely than conventional T cells, perhaps *via* suppressive activity of peripheral Tregs migrating back to the thymus (81). Aside from age-related changes in production, Treg activity is also altered with age. Functional changes in Tregs are very heterogenous and still require much work to fully understand. The controversies are well reviewed elsewhere (82), but the consensus is that aged Tregs are more suppressive and preferentially secrete IL-10 over TGF-β, which is preferred by young Tregs. This creates a rather complicated landscape balancing between being intensely regulatory while remaining systemically pro-inflammatory. Future studies should focus on the function of Tregs in various tissue niches with age, which may reveal more heterogeneity and may explain why the systemic view remains pro-inflammatory generally.

CD8 T cells likewise are deleteriously affected by aging. Decreased frequency of naïve cells (83) and narrowing of the TCR repertoire (84) have been known for many years now. During homeostasis, CD8 T cells undergo functional changes similar to CD4s. Especially in humans, peripheral CD8 T cells take on a suppressive phenotype resembling that of Foxp3+ CD4 Tregs (85). Because many of the age-related changes to CD8s are in the context of how they respond to pathogens, we will further discuss the effects of age on the function of CD8s in the next section.

A key feature of immune aging, especially in humans, is the inflation of the memory CD8 T cell compartment. Over time, virus specific CD8 memory cells predominate among total T cells in the periphery at homeostasis in older adults (86). In the past decade, much work has been done to categorize memory T cell subsets on the basis of surface marker expression and trafficking pattern. They can be broadly characterized as T central memory (Tcm), T effector memory (Tem), tissue resident memory T cells (Trm), and peripheral memory T cells (Tpm) (87). The expansion of memory T cells usually is found in the Tem compartment and has been mostly attributed to antigen stimulation by latent viral infections like cytomegalovirus (CMV) (88). In humans, these cells are typically described as Tem cells expressing CD45RA (TEMRA) (89). However these effects are not unique to CMV and they have also been linked to other common herpesviruses (90). These cells should not be conflated with exhausted T cells that can accumulate with chronic infections that result from consistently high levels of antigen presentation. Aged memory-inflated cells do not seem to follow an exhausted phenotype as defined by inhibitory receptor expression. TEMRA cells do not highly express PD-1 and they retain their effector function upon reactivation (91). Distinct from TEMRA cells, a recent report has described a novel subset of memory CD8 T cells that are associated with age (92). Using single cell RNA sequencing approaches, this novel subset, termed age associated T cells (Taa), uniquely express granzyme K which was associated with higher SASP factor secretion. They also found that the GzmK+ Taa cells expressed common exhaustion markers such as PD-1 and the transcription factor Tox. In contrast with many previous studies, this study did not observe increases in the TEMRA population. This reveals an emerging link between senescent cells and the exacerbation of immune aging which, with further work, may provide a means to rejuvenate some T cell responses with age.

#### 2.2.3 Immune-structural cell interactions

Structural cells like fibroblasts, endothelial and epithelial cells are likely the senescent cells present in the lung. Evidence for immune cell senescence remains controversial and dependent on the definitions of senescence. Some groups have identified subsets of senescent T cells in humans (93) while there is stronger evidence for senescent B cells, reviewed elsewhere (94). Interestingly, fewer studies have identified senescent T and B cells in mice, perhaps due to lack of consistent pathogen encounter. Senescent structural cells create two problems: 1) SASP production altering the tissue microenvironment and 2) morphological and functional changes in the structural cells. We’ve discussed at length the possible downstream effects of SASP production on the function of immune cells during homeostasis and will continue to touch upon how it affects responses to pathogens. Generally, proinflammatory SASP production can cause dysregulated responses in nearby immune cells simply by tonic cytokine signaling in the absence of an infection. When cells engage the senescence pathway, their morphology changes (95). When considering cells that maintain tight barriers in tissues like epithelial and endothelial cells, senescent morphological changes could result in loss of barrier integrity. While this has not yet been studied, it is likely that permeability in these barriers, especially endothelial barriers will result in improper trafficking of immune cells into the tissue and exacerbation of immunopathology. Future work should aim to address crosstalk between immune and structural cells and how senescence, *via* the two effects mentioned, may cause dysfunction in these communication networks. Certainly, in the context on an infection, all of these age and senescence-associated changes can prove to be deleterious during an infection.

### 2.3 The aged response to respiratory pathogens

In prior sections, we have discussed some of the myriad of ways age affects the homeostatic functions that are relevant for initiating and fostering immune responses to respiratory infections. Now we consider how these changes, including cellular senescence, integrate to leave older adults vulnerable to adverse outcomes following an infection. Influenza (flu) infection is of particular interest to our group and many others because it is among the greatest threats to older adults, who make up between 70 and 85% of flu related deaths (96). This includes individuals who succumb to secondary bacterial pneumonias, which we will also discuss. Many of these concepts also apply to SARS-CoV-2 infection and COVID-19 outcomes as well, which is well reviewed by others (97).

#### 2.3.1 The aged response to influenza infection

Work done by our group and many others has revealed a striking effect of age on outcomes of flu infection. This is particularly well characterized in mouse models, which have demonstrated that aged mice lose more body mass and are substantially delayed in the clearance of a sublethal flu infection when compared to young (98). There are numerous factors, many of which have been mentioned earlier, contributing to this deficit. The flu virion infects lung epithelial cells with high specificity (99). While not confirmed in the context of natural aging *in vivo*, epithelial cells are known to undergo senescence in response to radiation (100). It also has been suggested that viral infection itself can induce senescence: a recent study characterized a SASP phenotype that was pathogenic in the progression of COVID-19 and increased immunopathology (101). It is therefore possible that senescence can also be induced by flu infection.

The heightened inflammation with aging and SASP results in the aberrant function of many cell types already discussed. Resident alveolar macrophages are among the first responders to any respiratory pathogen, including flu. These cells are developed very early on in gestation and populate the lungs by responding to cytokines secreted by fetal lung epithelial cells (102, 103). With age, these cells begin to be replenished by bone marrow-derived monocytes rather than by self-renewal (104). A recent study in mice (105) examined the consequences of this in the context of flu infection and found that while fetal derived alveolar macrophages are able to self-renew effectively over time, they are outcompeted by monocyte-derived counterparts that are more glycolytic and proliferative. The monocyte-derived alveolar macrophages are in a more proinflammatory state during flu infection and cause immunopathology and prolong the course of infection, delaying adequate healing. While beyond the scope of this study, the consequences are likely not limited to a primary infection. If the wound healing phase of the antiviral response is limited, the generation of T and B cell memory is likely affected as well. Memory T cell differentiation is heavily reliant on TGF-β, a key molecule for effective wound healing (106). Therefore, this heightened inflammatory state can have downstream consequences outside of the activity of alveolar macrophages. As with alveolar macrophages, DCs are an integral part of mounting a robust flu response and are also affected by alterations in inflammatory states.

In order to initiate a robust antiviral response, type I interferons (IFN-α and -β) are secreted by infected cells as well as dendritic cells and macrophages/monocytes in response to exposure to viral components (107). Type I IFN signaling activates APCs and allows for effective antigen presentation to T cells. Importantly, it has been shown that in older adults, monocytes express less of the type I IFN signaling machinery (108) and that DCs do not effectively express costimulatory molecules (40, 41). Both of these factors could then contribute to reduced T cell priming and effective viral clearance (109).

When responding to an infection, CD4 T cells are critically important for providing help to CD8 T cells and B cells as well as mediating cytotoxic activity directly (110). A hallmark of CD4 T cells is their ability to differentiate into specialized T helper subsets that are more effective at directing responses towards specific classes of pathogens. We have discussed the changes during homeostasis in basal CD4 subset balance and now we turn to the various deficits in CD4 subsets during an aged response to flu infection. This topic has been a focus of our group for many years now and evidence is emerging that the senescent environment is a driving force in causing these deficits.

**Figure 1:**
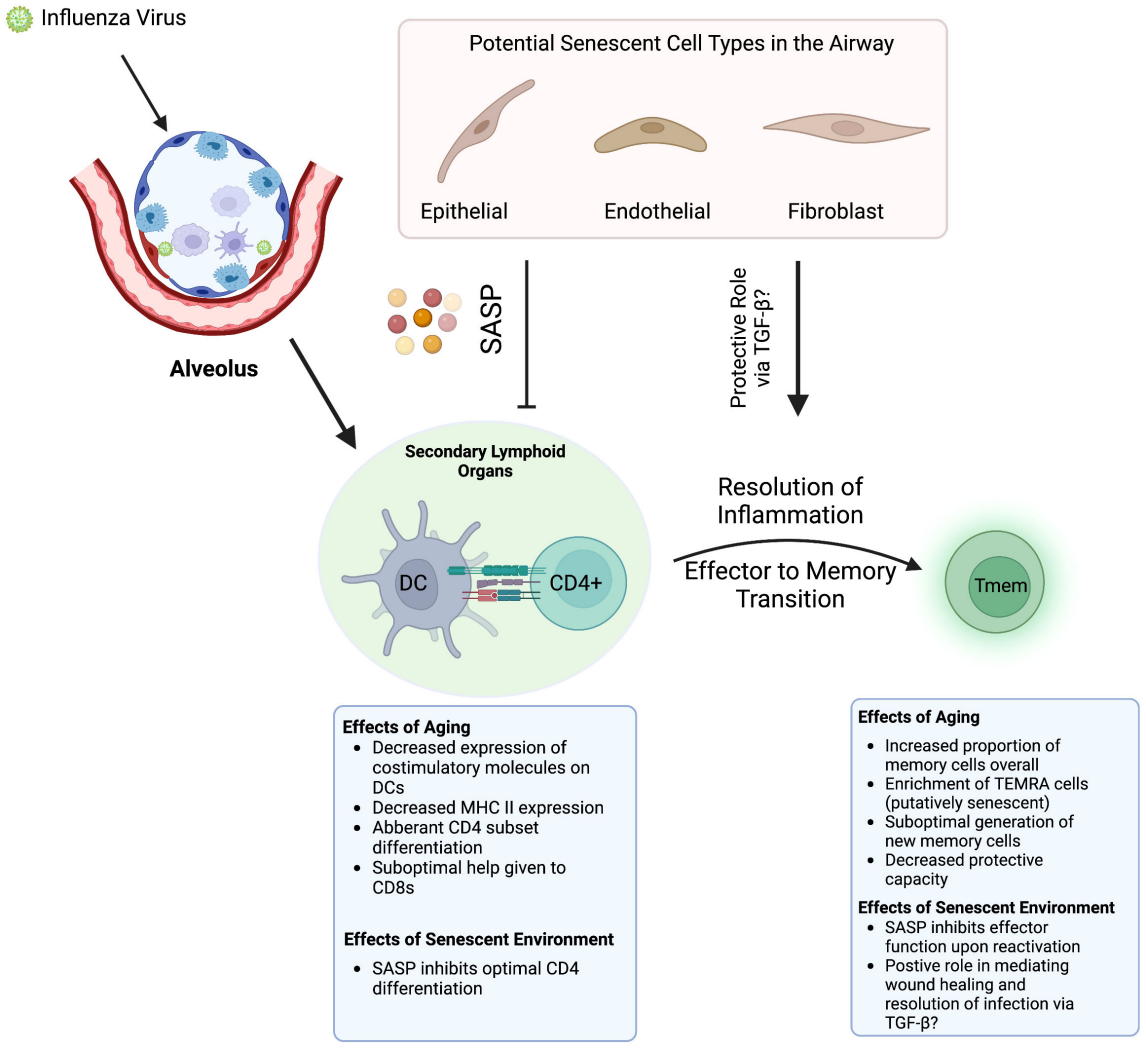
How Aging and Senescence Regulate Antiviral T Cell Responses. When a flu infection occurs, APCs such as DCs present antigen to naïve CD4 T cells, which requires costiumulation and a conducive cytokine milieu. Aging results in various defects in this process and senescent cells alter the milieu by secreting SASP factors. These perturbations result in suboptimal responses in both the CD4 and CD8 T cell compartments. Whenever the pathogen is cleared, the return to homeostasis and the effector to memory transition is also affected. Especially in the CD8 compartment, memory cells generated following infection are also deleteriously affected by aging. It is possible that SASP may play a role in regulating memory differentiation *via* its wound healing activities and secretion of TGF-β. Created with BioRender.com.

Work done by our group demonstrated that, in mice, some of the age-related deficits in CD4 T cell function during infection are cell extrinsic and thus regulated by the aged microenvironment (111). Transfer of young CD4 T cells into aged hosts results in reduced trafficking into secondary lymphoid organs, reduced efficacy of priming by APCs, and reduced differentiation in to Bcl-6-expressing T follicular helper (Tfh) subsets. Less robust Tfh differentiation will deleteriously affect the formation and function of germinal centers and coordination of antibody-mediated responses. While these experiments were performed a few years before cellular senescence was being implicated in chronic pathologies of aging like IPF, osteoporosis, Alzheimer’s, and cardiovascular disease (9, 112–114), A theory is emerging in which senescent cells may be at the root of the aged microenvironment that was driving deficits in CD4 function during infection (**Figure 1**).

CD8 T cells are also extremely important for mediating an effective antiviral response to flu however the ways in which CD8s are affected by age are more nuanced. In general, deficits in aged CD8 function during flu infection have been mostly attributed to decreased TCR repertoire diversity (84), the function of APCs (115), and increased proportion of Tregs in the CD4 compartment (116), all from mechanistic mouse studies. This would explain why transferring young CD8 T cells into aged hosts still results in deficits during an infection but when isolated, aged CD8s can function as well as their young counterparts *in vitro* (115). However, in light of reports pointing towards the accumulation of various dysfunctional age-associated CD8 subsets in humans like GzmK-expressing Taa and virtual memory CD8s, which do show some signs of cellular senescence (117), there remains much more to be done to understand how the CD8 compartment is affected directly by age across species and what can be done to potentiate their responses independent of other cells.

There is also an emerging role for nonconventional immune cell types and how they can affect immune responses with age. Innate lymphoid cells (ILCs) derive developmentally from the same common lymphoid progenitor cell as B and T cells but lack adaptive antigen receptors and function similar to CD4 subsets in secreting type I, II, or III cytokines and expressing Tbet, GATA3, or RORγT, respectively (118). Type II ILCs (ILC2s), have been linked most strongly to regulating responses in the lungs, especially viral infection as reviewed elsewhere (119). Studies have shown that following flu infection, ILC2 secretion of IL-33 causes an asthma-like phenotype in young mice (120). Other studies have similarly found that ILC2s can drive a fibrotic phenotype following pathogen challenge in mice and observed increased ILC2 frequency in lungs of human idiopathic fibrosis patients (121). In this sense, ILC2s mirror senescent cells in exacerbating pro-fibrotic phenotypes. A more recent study in aged mice revealed a broad heterogeneity in the ILC2 compartment in the lungs. Aging was associated with decreased overall frequency of ILC2s in the lungs and were more transcriptionally diverse than young ILC2s. Interestingly, transfer of young ILC2s into aged mice prior to influenza infection significantly enhanced protection against morbidity and mortality (122). The implication of this study has yet to be fully recapitulated in humans, but it is likely, as with most cell types discussed, that aging results in nuanced changes in ILC2 function that results in an overall decline in functionality following an infection.

A dysfunctional B cell response is another hallmark of an aged response to flu infection. Most of these changes are not flu-specific and are a result of the various homeostatic alterations to antibody production that we discussed previously. Importantly, most of these deficits have consequences following a subsequent encounter either following a primary infection or a vaccine response, which is the focus of the next section.

Taken together, these factors contribute to delayed viral clearance and could have serious effects on tissue repair, exacerbation of immunopathology and failure to return to homeostasis (98). For example, this is clearly illustrated by the various inflammation-driven adverse outcomes following COVID-19 infection in older adults (123). This failure is also one of the factors involved in the enhanced susceptibility of older adults to develop secondary bacterial pneumonia following an influenza infection. The mechanisms of susceptibility to bacterial infection are well reviewed elsewhere (124), but the main contributors are damage to the lung epithelium following flu infection as well as dysfunctional cytokine signaling by innate cells. Although we have mentioned various ways innate cells in the lung are affected by age, there have been few studies connecting flu infection, bacterial pneumonia, *and* the effects of age together. One study, utilizing a *Pseudomonas aeruginosa* pneumonia model demonstrated that aged mice, similar to what is overserved for older adults, were more severely affected by the bacterial infection after flu challenge (125). Clearly, more work is needed in this clinically important area.

## 3 Aged vaccine/memory responses

One goal of the generation of immunological memory is to reduce the pathology associated with a subsequent respiratory infection and limit lung damage. Importantly, apart from the primary response to infection, aging also diminishes the capacity of T and B cells to form durable and protective memory following either an infection or a vaccination. This is made evident by the fact that in spite of widespread and successful vaccination campaigns among older adults, they remain the most vulnerable group in terms of morbidity and mortality following infection. When considering how age is affecting the ability of older adults to effectively respond to an infection, immune memory is a key factor to consider, especially in the context of flu or COVID-19.

A key driver of dysfunctional memory responses in T cells, especially in older adults, is the reality of chronic herpesvirus infections such as cytomegalovirus, Epstein-Barr virus, and varicella (126). Estimates have ranged as high as 90% of individuals over 80 years age in developed countries are CMV positive (127). Although we have discussed how CMV status can affect memory cell populations over time, the consequences of this in terms of vaccination and infection is controversial. CMV-induced accumulation of CD4 memory T cells expressing a TEMRA phenotype was correlated with poor antibody production following flu vaccination in older adults (128). This highlights the central role of T cells in coordinating effective B cell responses to vaccination, which are also well reviewed elsewhere (129). A similar study found that CMV positive older adults who failed to respond to vaccination had higher expression of TNF-α and IL-6 (130). Conversely, a similarly structured study has found that CMV positivity was associated with heightened immune responses to vaccination in young individuals while CMV status did not correspond to any differences in the response among older participants (131). Mouse studies have found that while CMV diminishes the frequency of CD8 T cells responding to influenza, overall mortality is not affected (132, 133). There remains much to be understood regarding the role of CMV status in altering responses to infections or vaccinations, especially in humans. This may reveal some of the great heterogeneity seen with older populations (134) and some discrepancy could be attributed to differences in the cohorts utilized. and more studies should be performed to build consensus on this controversial but certainly relevant aspect of immune aging.

While CMV status may affect flu vaccine responses with age, there are many other factors that also contribute. CD4 T cell subsets, as with response to infection, are also deleteriously affected following vaccination. Work in our lab utilizing a crossprotective vaccine model against the internal flu nucleoprotein (NP) demonstrated that while vaccination improves excessive lung infiltration of innate inflammatory mediators, it fails to improve differentiation of CD4 T cells towards an antiviral Th1 polarized state in aged mice (135). This study also implicated a role for aged CD4 T cells in the susceptibility to secondary bacterial pneumonia since aged vaccinated mice remained severely vulnerable to mortality when coinfected with *Streptococcus pneumoniae*.

The effects of age on CD8 responses to flu vaccinations, especially in older adults, have been comprehensively reviewed elsewhere (136). Notably, the most reliable correlate of protection following flu vaccination in older adults can be found in the T cell compartment (137) and especially in the ability to generate IFN-γ preferentially over IL-10 upon stimulation (138). This observation is important for two reasons: (1) it demonstrates an easy and reliable correlate of protection that can be used to assess any proposed intervention, and (2) it demonstrates that interventions that target T cell responses may be the most beneficial for older adults.

Aging results in changes in the generation of memory T cells and we have shown that the ability of T cells from aged mice to generate fully functional memory T cells is significantly impacted (139). Furthermore, a recent study in mice demonstrated that increased establishment of flu-specific tissue resident T cells (Trms) following flu infection resulted in nonresolving immunopathology in the lungs of aged mice (140). Interestingly, this was linked to increased TGF-β (a known SASP factor) levels in the lung (8). While too much TGF-β may exacerbate this pathology, it is certainly required to form protective Trm cells. Thus, further work should elucidate how the dynamics of TGF-β signaling differ between young versus aged and how those differences result in either protective or pathogenic memory cell differentiation. In addition, future studies should also focus on how senescence impacts the generation of protective immune memory, including changes to the microenvironment that support the appropriate differentiation of memory T cells.

## 4 Role of senolytics in potentiating aged immune responses

Throughout this review, we have mentioned the various ways that senescent cells may influence the function of the immune system in response to infection and vaccination. As senescence emerges as a key driver of diseases of aging, we now have the tools to assess the impact of senescence on these age-related changes. One of the most effective tools is treatment with senolytics, which are drugs that can selectively target and induce apoptosis in senescent cells (141). The first senolytic treatment described was a combination treatment with dasatinib and quercetin (141) and is the most widely characterized. Others have also been identified including fisetin (142) and navitoclax (143). Generally, these drugs inhibit the anti-apoptotic pathways engaged by senescent cells thus inducing apoptosis. While these drugs have been shown in mouse studies to increase measures of healthspan and extend longevity (142, 144), very few studies have utilized this approach to ameliorate immune responses with age.

Recently, studies from our lab and others have begun to apply senolytic strategies to overcome age-related deficits during an infection. Our work with flu infection demonstrated that treatment of aged mice with D+Q decreased the proportion of Tregs in the lungs during infection in favor of a more healing GATA-3 expressing Th2 T cell population by mitigating the senescent environment (145). Strikingly, D+Q treatment completely abrogated TGF-β production in the lung following infection. Similar studies utilizing a model of nonspecific polymicrobial exposure concluded that treatment with fisetin in aged mice significantly protected them from mortality following microbial exposure *via* pet-store mice or their dirty bedding (146). While this study clearly implicates senescent cells play *some* role in age-related immune deficits to infection, more specialized experiments are needed to reveal exactly which arms of the immune system are sensitive to senolytic treatment.

Although the nuances of senolytics and immune responses have yet to be fully characterized in mechanistic pre-clinical animal studies, currently there are a number of clinical trials administering senolytics to older adults. D+Q has been demonstrated to be effective in clearing human senescent cells *in vivo* and improved symptoms of IPF in older adults (11, 147). Fisetin is being investigated in an ongoing trial to improve COVID-19 disease severity among nursing home residents (NCT04537299). The evidence that senescent cells are solely deleterious in modulating immune responses is far from clear. Some components of SASP, like TGF- β, can have consequences for the formation of memory T cells as previously discussed (106). The important role of senescent cells in mediating wound healing and the return to homeostasis cannot be ignored either (3). As the reports begin to emerge from these clinical trials, it is likely that the role of senescent cells in immune responses may be more complicated than previously appreciated.

## 5 Discussion

It is clear that aging has a significant impact on immune function, which can be detrimental when attempting to fight a respiratory infection. The root causes of these age-related changes are still not clear, but the accumulation of senescent cells likely plays a role. In the past, it has been quite difficult to identify truly senescent cells *in vivo* since this requires a preponderance of evidence to definitively determine that a cell is truly senescent. As mentioned earlier, this typically involves expression of senescence enforcement molecules p16 and/or p21, SA-β-Gal, SASP factors, and more. Nevertheless, new tools including transgenic mouse models (3, 148, 149) and the use of senolytic drugs (144) have recently enabled researchers to manipulate senescent cells *in vivo*. These tools will enable researchers to further investigate the role that senescent cells play in the lungs both at homeostasis and during an immune response. These studies will have the potential to move the field forward substantially and could result in novel approaches to improving immunity in older adults.

## Author contributions

BLT and LH both wrote, edited, and approved the final version of this manuscript.

## Funding

LH is supported by NIH grants P30AG067988 and R21AG071292. BLT is supported by NIH grant R21AG071292.

## Conflict of interest

The authors declare that the research was conducted in the absence of any commercial or financial relationships that could be construed as a potential conflict of interest.

## Publisher’s note

All claims expressed in this article are solely those of the authors and do not necessarily represent those of their affiliated organizations, or those of the publisher, the editors and the reviewers. Any product that may be evaluated in this article, or claim that may be made by its manufacturer, is not guaranteed or endorsed by the publisher.

## References

[1] Campisi J d'Adda di Fagagna F . Cellular senescence: When bad things happen to good cells. Nat Rev Mol Cell Biol (2007) 8(9):729–40. doi: 10.1038/nrm2233 17667954

[2] Wyld L Bellantuono I Tchkonia T Morgan J Turner O Foss F . Senescence and cancer: A review of clinical implications of senescence and senotherapies. Cancers (Basel) (2020) 12(8):2134. doi: 10.3390/cancers12082134 PMC7464619 32752135

[3] Demaria M Ohtani N Youssef SA Rodier F Toussaint W Mitchell JR . An essential role for senescent cells in optimal wound healing through secretion of pdgf-aa. Dev Cell (2014) 31(6):722–33. doi: 10.1016/j.devcel.2014.11.012 PMC4349629 25499914

[4] Khosla S Farr JN Tchkonia T Kirkland JL . The role of cellular senescence in ageing and endocrine disease. Nat Rev Endocrinol (2020) 16(5):263–75. doi: 10.1038/s41574-020-0335-y PMC7227781 32161396

[5] Alcorta DA Xiong Y Phelps D Hannon G Beach D Barrett JC . Involvement of the cyclin-dependent kinase inhibitor P16 (Ink4a) in replicative senescence of normal human fibroblasts. Proc Natl Acad Sci USA (1996) 93(24):13742–7. doi: 10.1073/pnas.93.24.13742 PMC19411 8943005

[6] Xue W Zender L Miething C Dickins RA Hernando E Krizhanovsky V . Senescence and tumour clearance is triggered by P53 restoration in murine liver carcinomas. Nature (2007) 445(7128):656–60. doi: 10.1038/nature05529 PMC4601097 17251933

[7] Hu L Li H Zi M Li W Liu J Yang Y . Why senescent cells are resistant to apoptosis: An insight for senolytic development. Front Cell Dev Biol (2022) 10:822816. doi: 10.3389/fcell.2022.822816 35252191 PMC8890612

[8] Acosta JC Banito A Wuestefeld T Georgilis A Janich P Morton JP . A complex secretory program orchestrated by the inflammasome controls paracrine senescence. Nat Cell Biol (2013) 15(8):978–90. doi: 10.1038/ncb2784 PMC3732483 23770676

[9] Schafer MJ White TA Iijima K Haak AJ Ligresti G Atkinson EJ . Cellular senescence mediates fibrotic pulmonary disease. Nat Commun (2017) 8:14532. doi: 10.1038/ncomms14532 28230051 PMC5331226

[10] Kellogg DL Musi N Nambiar AM . Cellular senescence in idiopathic pulmonary fibrosis. Curr Mol Biol Rep (2021) 7(3):31–40. doi: 10.1007/s40610-021-00145-4 34401216 PMC8358258

[11] Justice JN Nambiar AM Tchkonia T LeBrasseur NK Pascual R Hashmi SK . Senolytics in idiopathic pulmonary fibrosis: Results from a first-in-Human, open-label, pilot study. EBioMedicine (2019) 40:554–63. doi: 10.1016/j.ebiom.2018.12.052 PMC6412088 30616998

[12] Brandsma CA de Vries M Costa R Woldhuis RR Königshoff M Timens W . Lung ageing and copd: Is there a role for ageing in abnormal tissue repair? Eur Respir Rev (2017) 26(146):170073. doi: 10.1183/16000617.0073-2017 29212834 PMC9488745

[13] Ito K Barnes PJ . Copd as a disease of accelerated lung aging. Chest (2009) 135(1):173–80. doi: 10.1378/chest.08-1419 19136405

[14] Silverman EK Chapman HA Drazen JM Weiss ST Rosner B Campbell EJ . Genetic epidemiology of severe, early-onset chronic obstructive pulmonary disease. risk to relatives for airflow obstruction and chronic bronchitis. Am J Respir Crit Care Med (1998) 157(6 Pt 1):1770–8. doi: 10.1164/ajrccm.157.6.9706014 9620904

[15] Chung KF . Cytokines in chronic obstructive pulmonary disease. Eur Respir J Suppl (2001) 34:50s–9s. doi: 10.1183/09031936.01.00229701 12392035

[16] Donaldson GC Seemungal TA Patel IS Bhowmik A Wilkinson TM Hurst JR . Airway and systemic inflammation and decline in lung function in patients with copd. Chest (2005) 128(4):1995–2004. doi: 10.1378/chest.128.4.1995 16236847 PMC7172405

[17] Kiszałkiewicz JM Majewski S Piotrowski WJ Górski P Pastuszak-Lewandoska D Migdalska-Sęk M . Evaluation of selected Il6/Stat3 pathway molecules and mirna expression in chronic obstructive pulmonary disease. Sci Rep (2021) 11(1):22756. doi: 10.1038/s41598-021-01950-8 34815425 PMC8610981

[18] Coppé JP Patil CK Rodier F Sun Y Muñoz DP Goldstein J . Senescence-associated secretory phenotypes reveal cell-nonautonomous functions of oncogenic ras and the P53 tumor suppressor. PloS Biol (2008) 6(12):2853–68. doi: 10.1371/journal.pbio.0060301 PMC2592359 19053174

[19] Rashid K Sundar IK Gerloff J Li D Rahman I . Lung cellular senescence is independent of aging in a mouse model of Copd/Emphysema. Sci Rep (2018) 8(1):9023. doi: 10.1038/s41598-018-27209-3 29899396 PMC5998122

[20] Woldhuis RR de Vries M Timens W van den Berge M Demaria M Oliver BGG . Link between increased cellular senescence and extracellular matrix changes in copd. Am J Physiol Lung Cell Mol Physiol (2020) 319(1):L48–60. doi: 10.1152/ajplung.00028.2020 32460521

[21] Hogg JC Timens W . The pathology of chronic obstructive pulmonary disease. Annu Rev Pathol (2009) 4:435–59. doi: 10.1146/annurev.pathol.4.110807.092145 18954287

[22] Togo S Holz O Liu X Sugiura H Kamio K Wang X . Lung fibroblast repair functions in patients with chronic obstructive pulmonary disease are altered by multiple mechanisms. Am J Respir Crit Care Med (2008) 178(3):248–60. doi: 10.1164/rccm.200706-929OC PMC2542423 18467512

[23] Wilkinson HN Hardman MJ . Senescence in wound repair: Emerging strategies to target chronic healing wounds. Front Cell Dev Biol (2020) 8:773. doi: 10.3389/fcell.2020.00773 32850866 PMC7431694

[24] Schmitt V Rink L Uciechowski P . The Th17/Treg balance is disturbed during aging. Exp Gerontol (2013) 48(12):1379–86. doi: 10.1016/j.exger.2013.09.003 24055797

[25] Silva LEF Lourenço JD Silva KR Santana FPR Kohler JB Moreira AR . Th17/Treg imbalance in copd development: Suppressors of cytokine signaling and signal transducers and activators of transcription proteins. Sci Rep (2020) 10(1):15287. doi: 10.1038/s41598-020-72305-y 32943702 PMC7499180

[26] Provisional covid-19 death counts by age in years 2020-2022. In: National center for health statistics. Available at: https://data.cdc.gov/d/3apk-4u4f.

[27] Estimated influenza disease burden, by season — united states, 2010-11 through 2019-20 influenza seasons. In: Centers for disease control and prevention , national center for immunization and respiratory diseases (NCIRD). Available at: https://www.cdc.gov/flu/about/burden/past-seasons.html.

[28] Salminen A Kaarniranta K Kauppinen A . Inflammaging: Disturbed interplay between autophagy and inflammasomes. Aging (2012) 4(3):166–75. doi: 10.18632/aging.100444 PMC3348477 22411934

[29] Frasca D Blomberg BB . Inflammaging decreases adaptive and innate immune responses in mice and humans. Biogerontology (2016) 17(1):7–19. doi: 10.1007/s10522-015-9578-8 25921609 PMC4626429

[30] Franceschi C Bonafè M Valensin S Olivieri F De Luca M Ottaviani E . Inflamm-aging. An evolutionary perspective on immunosenescence. Ann N Y Acad Sci (2000) 908:244–54. doi: 10.1111/j.1749-6632.2000.tb06651.x 10911963

[31] Chang R Yee KL Sumbria RK . Tumor necrosis factor α inhibition for alzheimer's disease. J Cent Nerv Syst Dis (2017) 9:1179573517709278. doi: 10.1177/1179573517709278 28579870 PMC5436834

[32] Prattichizzo F De Nigris V Spiga R Mancuso E La Sala L Antonicelli R . Inflammageing and metaflammation: The yin and yang of type 2 diabetes. Ageing Res Rev (2018) 41:1–17. doi: 10.1016/j.arr.2017.10.003 29081381

[33] Rezuș E Cardoneanu A Burlui A Luca A Codreanu C Tamba BI . The link between inflammaging and degenerative joint diseases. Int J Mol Sci (2019) 20(3):614. doi: 10.3390/ijms20030614 PMC6386892 30708978

[34] Whiting CC Siebert J Newman AM Du H-W Alizadeh AA Goronzy J . Large-Scale and comprehensive immune profiling and functional analysis of normal human aging. PloS One (2015) 10(7):e0133627. doi: 10.1371/journal.pone.0133627 26197454 PMC4509650

[35] Sayed N Huang Y Nguyen K Krejciova-Rajaniemi Z Grawe AP Gao T . An inflammatory aging clock (Iage) based on deep learning tracks multimorbidity, immunosenescence, frailty and cardiovascular aging. Nat Aging (2021) 1(7):598–615. doi: 10.1038/s43587-021-00082-y 34888528 PMC8654267

[36] Claesson MJ Jeffery IB Conde S Power SE O'Connor EM Cusack S . Gut microbiota composition correlates with diet and health in the elderly. Nature (2012) 488(7410):178–84. doi: 10.1038/nature11319 22797518

[37] Conley MN Wong CP Duyck KM Hord N Ho E Sharpton TJ . Aging and serum mcp-1 are associated with gut microbiome composition in a murine model. PeerJ (2016) 4:e1854. doi: 10.7717/peerj.1854 27069796 PMC4824877

[38] Tilstra JS Clauson CL Niedernhofer LJ Robbins PD . Nf-κb in aging and disease. Aging Dis (2011) 2(6):449–65. PMC3295063 22396894

[39] Prakash S Agrawal S Vahed H Ngyuen M BenMohamed L BenMohamad L . Dendritic cells from aged subjects contribute to chronic airway inflammation by activating bronchial epithelial cells under steady state. Mucosal Immunol (2014) 7(6):1386–94. doi: 10.1038/mi.2014.28 PMC4205198 24759206

[40] Li G Smithey MJ Rudd BD Nikolich-Žugich J . Age-associated alterations in Cd8α+ dendritic cells impair Cd8 T-cell expansion in response to an intracellular bacterium. Aging Cell (2012) 11(6):968–77. doi: 10.1111/j.1474-9726.2012.00867.x PMC3533767 22862959

[41] Zacca ER Crespo MI Acland RP Roselli E Núñez NG Maccioni M . Aging impairs the ability of conventional dendritic cells to cross-prime Cd8+ T cells upon stimulation with a Tlr7 ligand. PloS One (2015) 10(10):e0140672. doi: 10.1371/journal.pone.0140672 26474053 PMC4608578

[42] Steger MM Maczek C Grubeck-Loebenstein B . Morphologically and functionally intact dendritic cells can be derived from the peripheral blood of aged individuals. Clin Exp Immunol (1996) 105(3):544–50. doi: 10.1046/j.1365-2249.1996.d01-790.x PMC2200540 8809147

[43] Agrawal A Agrawal S Cao JN Su H Osann K Gupta S . Altered innate immune functioning of dendritic cells in elderly humans: A role of phosphoinositide 3-Kinase-Signaling pathway. J Immunol (2007) 178(11):6912–22. doi: 10.4049/jimmunol.178.11.6912 17513740

[44] Lung TL Saurwein-Teissl M Parson W Schönitzer D Grubeck-Loebenstein B . Unimpaired dendritic cells can be derived from monocytes in old age and can mobilize residual function in senescent T cells. Vaccine (2000) 18(16):1606–12. doi: 10.1016/s0264-410x(99)00494-6 10689136

[45] Hall BM Balan V Gleiberman AS Strom E Krasnov P Virtuoso LP . Aging of mice is associated with P16(Ink4a)- and β-Galactosidase-Positive macrophage accumulation that can be induced in young mice by senescent cells. Aging (Albany NY) (2016) 8(7):1294–315. doi: 10.18632/aging.100991 PMC4993332 27391570

[46] Su L Dong Y Wang Y Guan B Lu Y Wu J . Potential role of senescent macrophages in radiation-induced pulmonary fibrosis. Cell Death Dis (2021) 12(6):527. doi: 10.1038/s41419-021-03811-8 34023858 PMC8141056

[47] Pan J Li D Xu Y Zhang J Wang Y Chen M . Inhibition of bcl-2/Xl with abt-263 selectively kills senescent type ii pneumocytes and reverses persistent pulmonary fibrosis induced by ionizing radiation in mice. Int J Radiat Oncol Biol Phys (2017) 99(2):353–61. doi: 10.1016/j.ijrobp.2017.02.216 PMC6853175 28479002

[48] Lin Y Xu Z . Fibroblast senescence in idiopathic pulmonary fibrosis. Front Cell Dev Biol (2020) 8:593283. doi: 10.3389/fcell.2020.593283 33324646 PMC7723977

[49] Álvarez D Cárdenes N Sellarés J Bueno M Corey C Hanumanthu VS . Ipf lung fibroblasts have a senescent phenotype. Am J Physiol Lung Cell Mol Physiol (2017) 313:L1164 – L1173. doi: 10.1152/ajplung.00220.2017 PMC6148001 28860144

[50] Papadopoulou A Kletsas D . Human lung fibroblasts prematurely senescent after exposure to ionizing radiation enhance the growth of malignant lung epithelial cells *in vitro* and *in vivo* . Int J Oncol (2011) 39(4):989–99. doi: 10.3892/ijo.2011.1132 21814715

[51] von Kobbe C . Targeting senescent cells: Approaches, opportunities, challenges. Aging (Albany NY) (2019) 11(24):12844–61. doi: 10.18632/aging.102557 PMC6949083 31789602

[52] Qian H Luo N Chi Y . Aging-shifted prostaglandin profile in endothelium as a factor in cardiovascular disorders. J Aging Res (2012) 2012:121390. doi: 10.1155/2012/121390 22500225 PMC3303603

[53] Zhao J Legge K Perlman S . Age-related increases in Pgd(2) expression impair respiratory dc migration, resulting in diminished T cell responses upon respiratory virus infection in mice. J Clin Invest (2011) 121(12):4921–30. doi: 10.1172/JCI59777 PMC3226008 22105170

[54] Zharhary D . Age-related changes in the capability of the bone marrow to generate b cells. J Immunol (1988) 141(6):1863–9. 3262642

[55] Blanco E Pérez-Andrés M Arriba-Méndez S Contreras-Sanfeliciano T Criado I Pelak O . Age-associated distribution of normal b-cell and plasma cell subsets in peripheral blood. J Allergy Clin Immunol (2018) 141(6):2208–19.e16. doi: 10.1016/j.jaci.2018.02.017 29505809

[56] Tabibian-Keissar H Hazanov L Schiby G Rosenthal N Rakovsky A Michaeli M . Aging affects b-cell antigen receptor repertoire diversity in primary and secondary lymphoid tissues. Eur J Immunol (2016) 46(2):480–92. doi: 10.1002/eji.201545586 26614343

[57] Muramatsu M Kinoshita K Fagarasan S Yamada S Shinkai Y Honjo T . Class switch recombination and hypermutation require activation-induced cytidine deaminase (Aid), a potential rna editing enzyme. Cell (2000) 102(5):553–63. doi: 10.1016/s0092-8674(00)00078-7 11007474

[58] Frasca D van der Put E Riley RL Blomberg BB . Reduced ig class switch in aged mice correlates with decreased E47 and activation-induced cytidine deaminase. J Immunol (2004) 172(4):2155–62. doi: 10.4049/jimmunol.172.4.2155 14764681

[59] Frasca D Landin AM Lechner SC Ryan JG Schwartz R Riley RL . Aging down-regulates the transcription factor E2a, activation-induced cytidine deaminase, and ig class switch in human b cells. J Immunol (2008) 180(8):5283–90. doi: 10.4049/jimmunol.180.8.5283 18390709

[60] Birjandi SZ Ippolito JA Ramadorai AK Witte PL . Alterations in marginal zone macrophages and marginal zone b cells in old mice. J Immunol (2011) 186(6):3441–51. doi: 10.4049/jimmunol.1001271 PMC3420341 21307289

[61] Masters AR Jellison ER Puddington L Khanna KM Haynes L . Attrition of T cell zone fibroblastic reticular cell number and function in aged spleens. Immunohorizons (2018) 2(5):155–63. doi: 10.4049/immunohorizons.1700062 PMC6350919 30706058

[62] Denton AE Dooley J Cinti I Silva-Cayetano A Fra-Bido S Innocentin S . Targeting Tlr4 during vaccination boosts madcam-1. Sci Immunol (2022) 7(71):eabk0018. doi: 10.1126/sciimmunol.abk0018 35522725 PMC7612953

[63] Rinaldi S Pallikkuth S George VK de Armas LR Pahwa R Sanchez CM . Paradoxical aging in hiv: Immune senescence of b cells is most prominent in young age. Aging (Albany NY) (2017) 9(4):1307–25. doi: 10.18632/aging.101229 PMC5425129 28448963

[64] Frasca D Diaz A Romero M Blomberg BB . Human peripheral Late/Exhausted memory b cells express a senescent-associated secretory phenotype and preferentially utilize metabolic signaling pathways. Exp Gerontol (2017) 87(Pt A):113–20. doi: 10.1016/j.exger.2016.12.001 27931848

[65] Hao Y O'Neill P Naradikian MS Scholz JL Cancro MP . A b-cell subset uniquely responsive to innate stimuli accumulates in aged mice. Blood (2011) 118(5):1294–304. doi: 10.1182/blood-2011-01-330530 PMC3152496 21562046

[66] Rubtsov AV Rubtsova K Fischer A Meehan RT Gillis JZ Kappler JW . Toll-like receptor 7 (Tlr7)-driven accumulation of a novel Cd11c^+^ b-cell population is important for the development of autoimmunity. Blood (2011) 118(5):1305–15. doi: 10.1182/blood-2011-01-331462 PMC3152497 21543762

[67] Saadoun D Terrier B Bannock J Vazquez T Massad C Kang I . Expansion of autoreactive unresponsive Cd21-/Low b cells in sjögren's syndrome-associated lymphoproliferation. Arthritis Rheum (2013) 65(4):1085–96. doi: 10.1002/art.37828 PMC4479193 23279883

[68] Moir S Ho J Malaspina A Wang W DiPoto AC O'Shea MA . Evidence for hiv-associated b cell exhaustion in a dysfunctional memory b cell compartment in hiv-infected viremic individuals. J Exp Med (2008) 205(8):1797–805. doi: 10.1084/jem.20072683 PMC2525604 18625747

[69] Johnson JL Rosenthal RL Knox JJ Myles A Naradikian MS Madej J . The transcription factor T-bet resolves memory b cell subsets with distinct tissue distributions and antibody specificities in mice and humans. Immunity (2020) 52(5):842–55.e6. doi: 10.1016/j.immuni.2020.03.020 32353250 PMC7242168

[70] Mouat IC Horwitz MS . Age-associated b cells in viral infection. PloS Pathog (2022) 18(3):e1010297. doi: 10.1371/journal.ppat.1010297 35298565 PMC8929649

[71] Palmer DB . The effect of age on thymic function. Front Immunol (2013) 4:316. doi: 10.3389/fimmu.2013.00316 24109481 PMC3791471

[72] Goronzy JJ Lee WW Weyand CM . Aging and T-cell diversity. Exp Gerontol (2007) 42(5):400–6. doi: 10.1016/j.exger.2006.11.016 PMC2680153 17218073

[73] Qi Q Liu Y Cheng Y Glanville J Zhang D Lee J-Y . Diversity and clonal selection in the human T-cell repertoire. Proc Natl Acad Sci (2014) 111(36):13139–44. doi: 10.1073/pnas.1409155111 PMC4246948 25157137

[74] Egorov ES Kasatskaya SA Zubov VN Izraelson M Nakonechnaya TO Staroverov DB . The changing landscape of naive T cell receptor repertoire with human aging. Front Immunol (2018) 9:1618. doi: 10.3389/fimmu.2018.01618 30087674 PMC6066563

[75] De Martinis M Modesti M Ginaldi L . Phenotypic and functional changes of circulating monocytes and polymorphonuclear leucocytes from elderly persons. Immunol Cell Biol (2004) 82(4):415–20. doi: 10.1111/j.0818-9641.2004.01242.x 15283852

[76] Bhadricha H Patel V Singh AK Savardekar L Patil A Surve S . Increased frequency of Th17 cells and il-17 levels are associated with low bone mineral density in postmenopausal women. Sci Rep (2021) 11(1):16155. doi: 10.1038/s41598-021-95640-0 34373550 PMC8352954

[77] Zhu L Hua F Ding W Ding K Zhang Y Xu C . The correlation between the Th17/Treg cell balance and bone health. Immun Ageing (2020) 17:30. doi: 10.1186/s12979-020-00202-z 33072163 PMC7557094

[78] Ouyang X Yang Z Zhang R Arnaboldi P Lu G Li Q . Potentiation of Th17 cytokines in aging process contributes to the development of colitis. Cell Immunol (2011) 266(2):208–17. doi: 10.1016/j.cellimm.2010.10.007 PMC3006034 21074754

[79] Faust HJ Zhang H Han J Wolf MT Jeon OH Sadtler K . Il-17 and immunologically induced senescence regulate response to injury in osteoarthritis. J Clin Invest (2020) 130(10):5493–507. doi: 10.1172/JCI134091 PMC7524483 32955487

[80] Sakaguchi S Yamaguchi T Nomura T Ono M . Regulatory T cells and immune tolerance. Cell (2008) 133(5):775–87. doi: 10.1016/j.cell.2008.05.009 18510923

[81] Thiault N Darrigues J Adoue V Gros M Binet B Perals C . Peripheral regulatory T lymphocytes recirculating to the thymus suppress the development of their precursors. Nat Immunol (2015) 16(6):628–34. doi: 10.1038/ni.3150 25939024

[82] Rocamora-Reverte L Melzer FL Würzner R Weinberger B . The complex role of regulatory T cells in immunity and aging. Front Immunol (2020) 11:616949. doi: 10.3389/fimmu.2020.616949 33584708 PMC7873351

[83] Cicin-Sain L Messaoudi I Park B Currier N Planer S Fischer M . Dramatic increase in naive T cell turnover is linked to loss of naive T cells from old primates. Proc Natl Acad Sci USA (2007) 104(50):19960–5. doi: 10.1073/pnas.0705905104 PMC2148405 18056811

[84] Ahmed M Lanzer KG Yager EJ Adams PS Johnson LL Blackman MA . Clonal expansions and loss of receptor diversity in the naive Cd8 T cell repertoire of aged mice. J Immunol (2009) 182(2):784–92. doi: 10.4049/jimmunol.182.2.784 PMC2724652 19124721

[85] Simone R Zicca A Saverino D . The frequency of regulatory Cd3+Cd8+Cd28- Cd25+ T lymphocytes in human peripheral blood increases with age. J Leukoc Biol (2008) 84(6):1454–61. doi: 10.1189/jlb.0907627 18780874

[86] O'Hara GA Welten SP Klenerman P Arens R . Memory T cell inflation: Understanding cause and effect. Trends Immunol (2012) 33(2):84–90. doi: 10.1016/j.it.2011.11.005 22222196

[87] Martin MD Badovinac VP . Defining memory Cd8 T cell. Front Immunol (2018) 9:2692. doi: 10.3389/fimmu.2018.02692 30515169 PMC6255921

[88] Holtappels R Pahl-Seibert MF Thomas D Reddehase MJ . Enrichment of immediate-early 1 (M123/Pp89) peptide-specific Cd8 T cells in a pulmonary Cd62l(Lo) memory-effector cell pool during latent murine cytomegalovirus infection of the lungs. J Virol (2000) 74(24):11495–503. doi: 10.1128/jvi.74.24.11495-11503.2000 PMC112429 11090146

[89] Wertheimer AM Bennett MS Park B Uhrlaub JL Martinez C Pulko V . Aging and cytomegalovirus infection differentially and jointly affect distinct circulating T cell subsets in humans. J Immunol (2014) 192(5):2143–55. doi: 10.4049/jimmunol.1301721 PMC3989163 24501199

[90] Lang A Brien JD Nikolich-Zugich J . Inflation and long-term maintenance of Cd8 T cells responding to a latent herpesvirus depend upon establishment of latency and presence of viral antigens. J Immunol (2009) 183(12):8077–87. doi: 10.4049/jimmunol.0801117 PMC4161222 20007576

[91] Akbar AN Henson SM . Are senescence and exhaustion intertwined or unrelated processes that compromise immunity? Nat Rev Immunol (2011) 11(4):289–95. doi: 10.1038/nri2959 21436838

[92] Mogilenko DA Shpynov O Andhey PS Arthur L Swain A Esaulova E . Comprehensive profiling of an aging immune system reveals clonal gzmk. Immunity (2021) 54(1):99–115.e12. doi: 10.1016/j.immuni.2020.11.005 33271118

[93] Martínez-Zamudio RI Dewald HK Vasilopoulos T Gittens-Williams L Fitzgerald-Bocarsly P Herbig U . Senescence-associated β-galactosidase reveals the abundance of senescent Cd8+ T cells in aging humans. Aging Cell (2021) 20(5):e13344. doi: 10.1111/acel.13344 33939265 PMC8135084

[94] Frasca D . Senescent b cells in aging and age-related diseases: Their role in the regulation of antibody responses. Exp Gerontol (2018) 107:55–8. doi: 10.1016/j.exger.2017.07.002 PMC5754260 28687479

[95] Cho KA Ryu SJ Oh YS Park JH Lee JW Kim H-P . Morphological adjustment of senescent cells by modulating caveolin-1 status*. J Biol Chem (2004) 279(40):42270–8. doi: 10.1074/jbc.M402352200 15263006

[96] [Dataset] estimated influenza disease burden, by season — united states, 2010-11 through 2019-20 influenza seasons . Available at: https://www.cdc.gov/flu/about/burden/past-seasons.html.

[97] Lynch SM Guo G Gibson DS Bjourson AJ Rai TS . Role of senescence and aging in sars-Cov-2 infection and covid-19 disease. Cells (2021) 10(12):3367. doi: 10.3390/cells10123367 34943875 PMC8699414

[98] Bartley JM Pan SJ Keilich SR Hopkins JW Al-Naggar IM Kuchel GA . Aging augments the impact of influenza respiratory tract infection on mobility impairments, muscle-localized inflammation, and muscle atrophy. Aging (Albany NY) (2016) 8(4):620–35. doi: 10.18632/aging.100882 PMC4925818 26856410

[99] Kuiken T Taubenberger JK . Pathology of human influenza revisited. Vaccine (2008) 26 Suppl 4:D59–66. doi: 10.1016/j.vaccine.2008.07.025 PMC2605683 19230162

[100] Hansel C Jendrossek V Klein D . Cellular senescence in the lung: The central role of senescent epithelial cells. Int J Mol Sci (2020) 21(9):3279. doi: 10.3390/ijms21093279 PMC7247355 32384619

[101] Lee S Yu Y Trimpert J Benthani F Mairhofer M Richter-Pechanska P . Virus-induced senescence is a driver and therapeutic target in covid-19. Nature (2021) 599(7884):283–9. doi: 10.1038/s41586-021-03995-1 34517409

[102] Schneider C Nobs SP Kurrer M Rehrauer H Thiele C Kopf M . Induction of the nuclear receptor ppar-Γ by the cytokine gm-csf is critical for the differentiation of fetal monocytes into alveolar macrophages. Nat Immunol (2014) 15(11):1026–37. doi: 10.1038/ni.3005 25263125

[103] Guilliams M De Kleer I Henri S Post S Vanhoutte L De Prijck S . Alveolar macrophages develop from fetal monocytes that differentiate into long-lived cells in the first week of life *via* gm-csf. J Exp Med (2013) 210(10):1977–92. doi: 10.1084/jem.20131199 PMC3782041 24043763

[104] Misharin AV Morales-Nebreda L Reyfman PA Cuda CM Walter JM McQuattie-Pimentel AC . Monocyte-derived alveolar macrophages drive lung fibrosis and persist in the lung over the life span. J Exp Med (2017) 214(8):2387–404. doi: 10.1084/jem.20162152 PMC5551573 28694385

[105] Li F Piattini F Pohlmeier L Feng Q Rehrauer H Kopf M . Monocyte-derived alveolar macrophages autonomously determine severe outcome of respiratory viral infection. Sci Immunol (2022) 7(73):eabj5761. doi: 10.1126/sciimmunol.abj5761 35776802

[106] Ma C Zhang N . Transforming growth factor-β signaling is constantly shaping memory T-cell population. Proc Natl Acad Sci USA (2015) 112(35):11013–7. doi: 10.1073/pnas.1510119112 PMC4568277 26283373

[107] Honda K Yanai H Takaoka A Taniguchi T . Regulation of the type I ifn induction: A current view. Int Immunol (2005) 17(11):1367–78. doi: 10.1093/intimm/dxh318 16214811

[108] Molony RD Nguyen JT Kong Y Montgomery RR Shaw AC Iwasaki A . Aging impairs both primary and secondary rig-I signaling for interferon induction in human monocytes. Sci Signal (2017) 10(509):eaan2392. doi: 10.1126/scisignal.aan2392 29233916 PMC6429941

[109] Feng E Balint E Poznanski SM Ashkar AA Loeb M . Aging and interferons: Impacts on inflammation and viral disease outcomes. Cells (2021) 10(3):708. doi: 10.3390/cells10030708 33806810 PMC8004738

[110] Sant AJ DiPiazza AT Nayak JL Rattan A Richards KA . Cd4 T cells in protection from influenza virus: Viral antigen specificity and functional potential. Immunol Rev (2018) 284(1):91–105. doi: 10.1111/imr.12662 29944766 PMC6070306

[111] Lefebvre JS Maue AC Eaton SM Lanthier PA Tighe M Haynes L . The aged microenvironment contributes to the age-related functional defects of Cd4 T cells in mice. Aging Cell (2012) 11(5):732–40. doi: 10.1111/j.1474-9726.2012.00836.x PMC3444657 22607653

[112] Farr JN Xu M Weivoda MM Monroe DG Fraser DG Onken JL . Targeting cellular senescence prevents age-related bone loss in mice. Nat Med (2017) 23(9):1072–9. doi: 10.1038/nm.4385 PMC5657592 28825716

[113] Musi N Valentine JM Sickora KR Baeuerle E Thompson CS Shen Q . Tau protein aggregation is associated with cellular senescence in the brain. Aging Cell (2018) 17(6):e12840. doi: 10.1111/acel.12840 30126037 PMC6260915

[114] Roos CM Zhang B Palmer AK Ogrodnik MB Pirtskhalava T Thalji NM . Chronic senolytic treatment alleviates established vasomotor dysfunction in aged or atherosclerotic mice. Aging Cell (2016) 15(5):973–7. doi: 10.1111/acel.12458 PMC5013022 26864908

[115] Jiang J Bennett AJ Fisher E Williams-Bey Y Shen H Murasko DM . Limited expansion of virus-specific Cd8 T cells in the aged environment. Mech Ageing Dev (2009) 130(11-12):713–21. doi: 10.1016/j.mad.2009.08.007 PMC2839881 19744506

[116] Williams-Bey Y Jiang J Murasko DM . Expansion of regulatory T cells in aged mice following influenza infection. Mech Ageing Dev (2011) 132(4):163–70. doi: 10.1016/j.mad.2011.03.001 PMC3111029 21414341

[117] Quinn KM Fox A Harland KL Russ BE Li J Nguyen THO . Age-related decline in primary Cd8 + t cell responses is associated with the development of senescence in virtual memory Cd8 + t cells. Cell Rep (2018) 23(12):3512–24. doi: 10.1016/j.celrep.2018.05.057 29924995

[118] Vivier E Artis D Colonna M Diefenbach A Di Santo JP Eberl G . Innate lymphoid cells: 10 years on. Cell (2018) 174(5):1054–66. doi: 10.1016/j.cell.2018.07.017 30142344

[119] Fonseca W Lukacs NW Elesela S Malinczak C-A . Role of Ilc2 in viral-induced lung pathogenesis. Front Immunol (2021) 12:675169. doi: 10.3389/fimmu.2021.675169 33953732 PMC8092393

[120] Chang Y-J Kim HY Albacker LA Baumgarth N McKenzie ANJ Smith DE . Innate lymphoid cells mediate influenza-induced airway hyper-reactivity independently of adaptive immunity. Nat Immunol (2011) 12(7):631–8. doi: 10.1038/ni.2045 PMC3417123 21623379

[121] Hams E Armstrong ME Barlow JL Saunders SP Schwartz C Cooke G . Il-25 and type 2 innate lymphoid cells induce pulmonary fibrosis. Proc Natl Acad Sci (2014) 111(1):367–72. doi: 10.1073/pnas.1315854111 PMC3890791 24344271

[122] D'Souza SS Shen X Fung ITH Ye L Kuentzel M Chittur SV . Compartmentalized effects of aging on group 2 innate lymphoid cell development and function. Aging Cell (2019) 18(6):e13019. doi: 10.1111/acel.13019 31429526 PMC6826140

[123] Bartleson JM Radenkovic D Covarrubias AJ Furman D Winer DA Verdin E . Sars-Cov-2, covid-19 and the ageing immune system. Nat Aging (2021) 1(9):769–82. doi: 10.1038/s43587-021-00114-7 PMC8570568 34746804

[124] Metzger DW Sun K . Immune dysfunction and bacterial coinfections following influenza. J Immunol (2013) 191(5):2047–52. doi: 10.4049/jimmunol.1301152 PMC3760235 23964104

[125] Cross RW Betancourt AM Schur MJ Norton EB Roy D Hartley T . Impact of primary influenza infection on the immune response to secondary bacterial infection in aged mice. Influenza Other Respir Viruses (2011) 5(Suppl 1):198–201. PMC4172348 29415086

[126] Pawelec G Akbar A Caruso C Effros R Grubeck-Loebenstein B Wikby A . Is immunosenescence infectious? Trends Immunol (2004) 25(8):406–10. doi: 10.1016/j.it.2004.05.006 15275638

[127] Arens R Remmerswaal EB Bosch JA van Lier RA . 5(Th) international workshop on cmv and immunosenescence - a shadow of cytomegalovirus infection on immunological memory. Eur J Immunol (2015) 45(4):954–7. doi: 10.1002/eji.201570044 25857239

[128] Derhovanessian E Theeten H Hähnel K Van Damme P Cools N Pawelec G . Cytomegalovirus-associated accumulation of late-differentiated Cd4 T-cells correlates with poor humoral response to influenza vaccination. Vaccine (2013) 31(4):685–90. doi: 10.1016/j.vaccine.2012.11.041 23196209

[129] Frasca D Blomberg BB . Aging induces b cell defects and decreased antibody responses to influenza infection and vaccination. Immun Ageing (2020) 17(1):37. doi: 10.1186/s12979-020-00210-z 33292323 PMC7674578

[130] Trzonkowski P Myśliwska J Szmit E Wieckiewicz J Lukaszuk K Brydak LB . Association between cytomegalovirus infection, enhanced proinflammatory response and low level of anti-hemagglutinins during the anti-influenza vaccination–an impact of immunosenescence. Vaccine (2003) 21(25-26):3826–36. doi: 10.1016/s0264-410x(03)00309-8 12922116

[131] Furman D Jojic V Sharma S Shen-Orr SS L. Angel CJ Onengut-Gumuscu S . Cytomegalovirus infection enhances the immune response to influenza. Sci Trans Med (2015) 7(281):281ra43–ra43. doi: 10.1126/scitranslmed.aaa2293 PMC4505610 25834109

[132] Cicin-Sain L Brien JD Uhrlaub JL Drabig A Marandu TF Nikolich-Zugich J . Cytomegalovirus infection impairs immune responses and accentuates T-cell pool changes observed in mice with aging. PloS Pathog (2012) 8(8):e1002849. doi: 10.1371/journal.ppat.1002849 22916012 PMC3420928

[133] Marandu TF Oduro JD Borkner L Dekhtiarenko I Uhrlaub JL Drabig A . Immune protection against virus challenge in aging mice is not affected by latent herpesviral infections. J Virol (2015) 89(22):11715–7. doi: 10.1128/jvi.01989-15 PMC4645637 26339051

[134] Nguyen QD Moodie EM Forget MF Desmarais P Keezer MR Wolfson C . Health heterogeneity in older adults: Exploration in the Canadian longitudinal study on aging. J Am Geriatrics Soc (2021) 69(3):678–87. doi: 10.1111/jgs.16919 33155270

[135] Lefebvre JS Lorenzo EC Masters AR Hopkins JW Eaton SM Smiley ST . Vaccine efficacy and T helper cell differentiation change with aging. Oncotarget (2016) 7(23):33581–94. doi: 10.18632/oncotarget.9254 PMC5085104 27177221

[136] McElhaney JE Verschoor CP Andrew MK Haynes L Kuchel GA Pawelec G . The immune response to influenza in older humans: Beyond immune senescence. Immun Ageing (2020) 17:10. doi: 10.1186/s12979-020-00181-1 32399058 PMC7204009

[137] McElhaney JE Xie D Hager WD Barry MB Wang Y Kleppinger A . T Cell responses are better correlates of vaccine protection in the elderly. J Immunol (2006) 176(10):6333–9. doi: 10.4049/jimmunol.176.10.6333 16670345

[138] McElhaney JE Ewen C Zhou X Kane KP Xie D Hager WD . Granzyme b: Correlates with protection and enhanced ctl response to influenza vaccination in older adults. Vaccine (2009) 27(18):2418–25. doi: 10.1016/j.vaccine.2009.01.136 PMC2800816 19368783

[139] Haynes L Eaton SM Burns EM Randall TD Swain SL . Cd4 T cell memory derived from young naive cells functions well into old age, but memory generated from aged naive cells functions poorly. Proc Natl Acad Sci USA (2003) 100(25):15053–8. doi: 10.1073/pnas.2433717100 PMC299903 14657384

[140] Goplen NP Wu Y Son YM Li C Wang Z Cheon IS . Tissue-resident Cd8+ T cells drive age-associated chronic lung sequelae after viral pneumonia. Sci Immunol (2020) 5(53):eabc4557. doi: 10.1126/sciimmunol.abc4557 33158975 PMC7970412

[141] Zhu Y Tchkonia T Pirtskhalava T Gower AC Ding H Giorgadze N . The achilles’ heel of senescent cells: From transcriptome to senolytic drugs. Aging Cell (2015) 14(4):644–58. doi: 10.1111/acel.12344 PMC4531078 25754370

[142] Yousefzadeh MJ Zhu Y McGowan SJ Angelini L Fuhrmann-Stroissnigg H Xu M . Fisetin is a senotherapeutic that extends health and lifespan. EBioMedicine (2018) 36:18–28. doi: 10.1016/j.ebiom.2018.09.015 30279143 PMC6197652

[143] Chang J Wang Y Shao L Laberge R-M Demaria M Campisi J . Clearance of senescent cells by Abt263 rejuvenates aged hematopoietic stem cells in mice. Nat Med (2016) 22(1):78–83. doi: 10.1038/nm.4010 26657143 PMC4762215

[144] Xu M Pirtskhalava T Farr JN Weigand BM Palmer AK Weivoda MM . Senolytics improve physical function and increase lifespan in old age. Nat Med (2018) 24(8):1246–56. doi: 10.1038/s41591-018-0092-9 PMC6082705 29988130

[145] Lorenzo EC Torrance BL Keilich SR Al-Naggar I Harrison A Xu M . Senescence-induced changes in Cd4 T cell differentiation can be alleviated by treatment with senolytics. Aging Cell (2022) 21(1):e13525. doi: 10.1111/acel.13525 34962049 PMC8761018

[146] Camell CD Yousefzadeh MJ Zhu Y Prata LGPL Huggins MA Pierson M . Senolytics reduce coronavirus-related mortality in old mice. Science (2021) 373(6552):eabe4832. doi: 10.1126/science.abe4832 34103349 PMC8607935

[147] Hickson LJ Langhi Prata LGP Bobart SA Evans TK Giorgadze N Hashmi SK . Senolytics decrease senescent cells in humans: Preliminary report from a clinical trial of dasatinib plus quercetin in individuals with diabetic kidney disease. EBioMedicine (2019) 47:446–56. doi: 10.1016/j.ebiom.2019.08.069 PMC6796530 31542391

[148] Wang B Wang L Gasek NS Zhou Y Kim T Guo C . An inducible *p21*-cre mouse model to monitor and manipulate p21-Highly-Expressing senescent cel*ls *in vivo. Nat Aging (2021) 1(10):962–73. doi: 10.1038/s43587-021-00107-6 PMC8746571 35024619

[149] Baker DJ Wijshake T Tchkonia T LeBrasseur NK Childs BG van de Sluis B . Clearance of P16ink4a-positive senescent cells delays ageing-associated disorders. Nature (2011) 479(7372):232–6. doi: 10.1038/nature10600 PMC3468323 22048312

